# A case series of a rare tendon rupture

**DOI:** 10.1093/jscr/rjab058

**Published:** 2021-03-29

**Authors:** Sabrina Weber, Bernd Zimmermann, Gian Bühler, Philipp F Stillhard

**Affiliations:** Department of Orthopaedic Surgery, Kantonsspital Graubünden, 7000 Chur, Switzerland; Department of Orthopaedic Surgery, Kantonsspital Graubünden, 7000 Chur, Switzerland; Department of Orthopaedic Surgery, Kantonsspital Graubünden, 7000 Chur, Switzerland; Department of Orthopaedic Surgery, Kantonsspital Graubünden, 7000 Chur, Switzerland

## Abstract

The distal triceps tendon rupture is a rare finding. Only 1% of tendon ruptures are related to it. The triceps brachii muscle has three parts. All of them insert together at the posterior surface of olecranon. Mostly, the tendon ruptured at this level of insertion. The typically trauma mechanism is a fall on the hand with fully extended elbow or a direct trauma. There are also some cases described after weightlifting or secondary due to insufficiency after total joint replacement of the elbow. The diagnosis is based on clinical findings. Ultrasound or magnetic resonance imaging diagnostic is secondary but might help to differentiate between partial or complete rupture as well as to assess tendon retraction. The diagnosis should be treated operatively. Until today, there is no standard of art of surgery techniques. We describe three cases with traumatic triceps tendon rupture fixed by a transosseous refixation.

## INTRODUCTION

### Case report

#### Patient history

Patient 1 was a 29-year-old man who was transferred to our emergency department from a corresponding hospital. There he was presented with severe pain at the left elbow and loss of muscle strength after lifting a 170 kg over his head. Furthermore, the patient was healthy with no further diseases.

Patient 2 was a 73-year-old man, who’s fallen on his extended left arm. After presenting by a general practitioner, he was transferred to our hospital for magnetic resonance imaging (MRI) diagnosis; a total triceps tendon rupture was diagnosed and the indication for surgery was set.

Patient 3 was a 56-year-old man, who has two distortions of the right elbow within 1 month. After the second distortion, he suffered from severe pain and a loss of strength as well as swelling of the right elbow. The clinical suspected diagnosis of a triceps tendon rupture was verified by MRI. The patient himself has several more medical conditions, such as coronary problems, diabetes and chronic pain at both knees and his chest.

#### Status and findings

In all three patients, the affected elbow was swollen and painful. Each of them had a significant loss of muscle strength and a reduced range of motion on the diseased elbow.

All of them were in general good condition, and none of them had any suspicion for infection. All three patients had a conventional radiography to exclude a fracture. In one of them, a so called ‘flake of bone-sign’ was seen; the other two were without any pathological findings.

**Figure 1:**
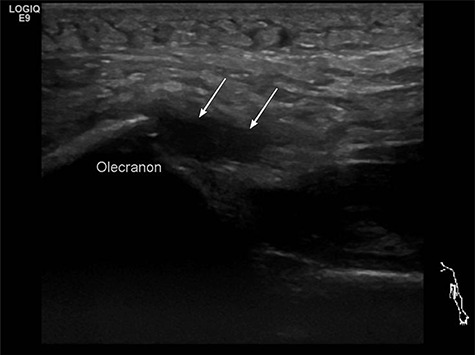
Preoperative ultrasound diagnostic.

In two patients, an additional MRI to verify the diagnosis was made. In both cases, MRI was ordered by the family doctor before transferring the patients to us. The third patient received an ultrasound sonography, where the rupture could be proofed (Fig. 1).

#### Therapy

In all three cases, we performed surgery with transosseous repair technique of the distal triceps tendon to the olecranon. In two patients, the surgery was performed in prone position with general anesthesia, and in one patient in supine position because he wished regional anesthesia.

### Operation technique

In all of the three cases described above we used the technique described by Steffes [1].

A direct approach to the olecranon and distal upper arm was performed.

**Figure 2:**
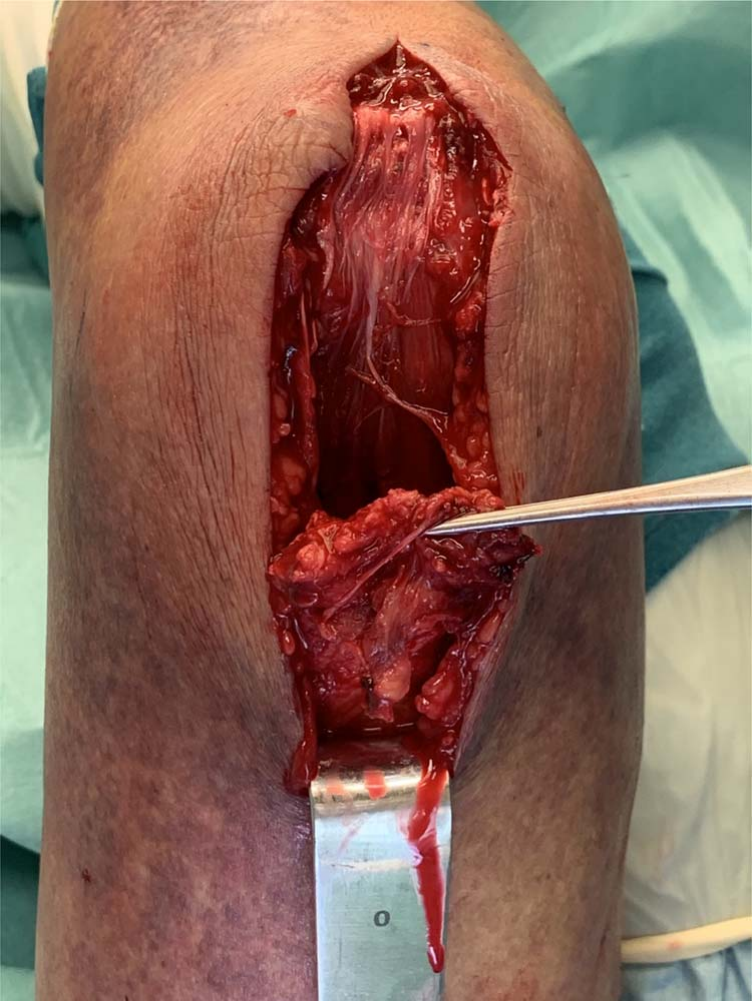
Intraoperative tendon rupture.

The ruptured tendon was visualized and sparingly debrided (Fig. 2). The original footprint of the tendon was cleaned with a sharp spoon.

Afterwards, we drilled two parallel holes through the olecranon with a 2.5-mm drill from the tip of the Olecranon to the distal dorsal ulna. We took care to leave enough space to the dorsal corticalis to prevent a tear out of the bone tunnels.

**Figure 3:**
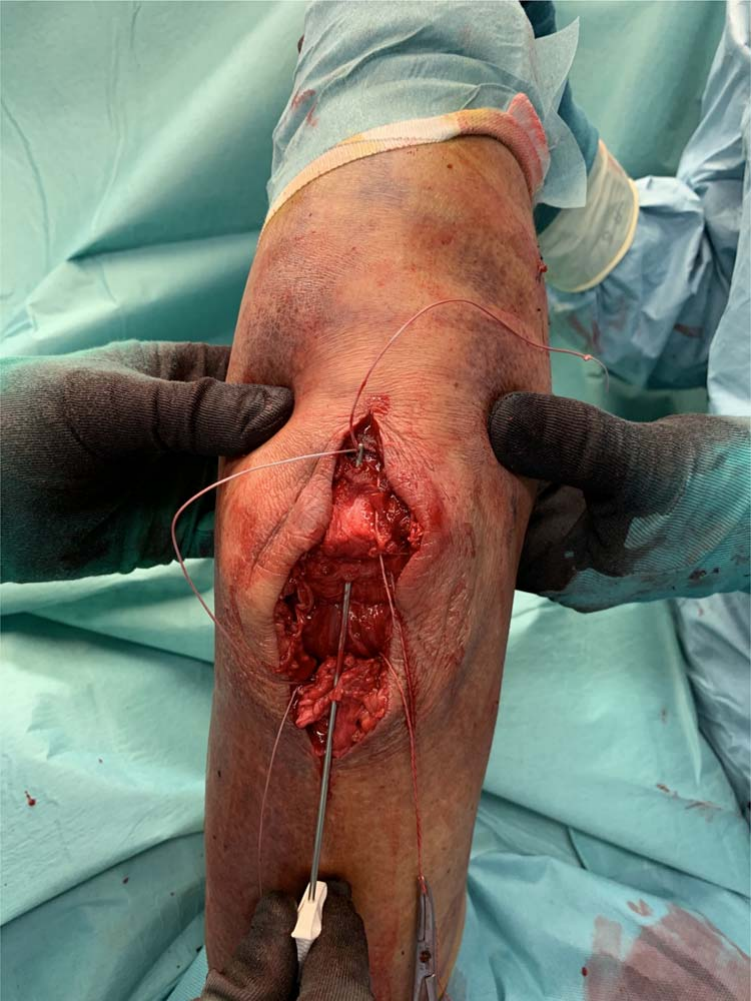
Intraoperative transosseous refixation.

The tendon was armed with two FiberWire sutures and one thread end of each FiberWire was passed through the bone tunnel from distal to proximal with a suture passing device (Fig. 3).

**Figure 4:**
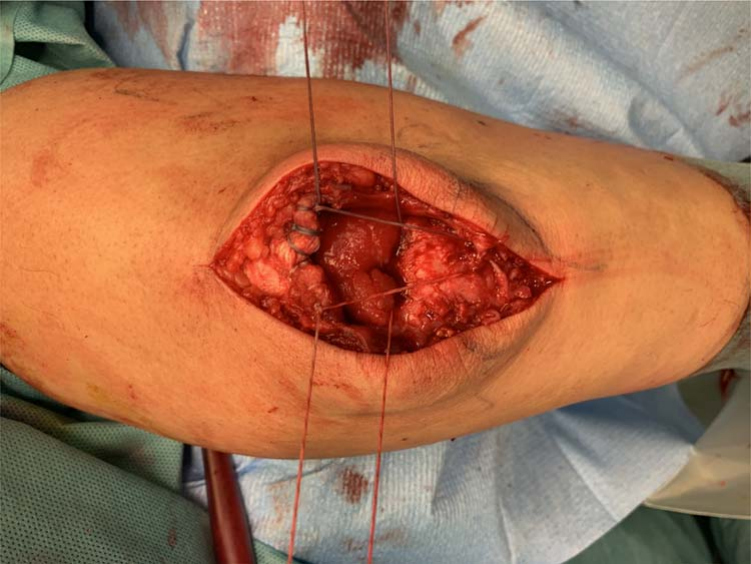
Intraoperative after transosseous refixation.

The tendon was then pulled in the original position and the threads were tightened and knotted. With the two-armed threads, we performed an adaption of the tendon so that it was well remodeled (Fig. 4). The tendon tension was controlled directly after the knotting and a flexion of almost 90° was reached.

The wound was then closed with subcutaneous sutures and a nonresorbable skin suture.

#### After care

All patients were initially immobilized with a splint and received a special cast after 5 days. The limitation of range of movement was a flexion–restriction of maximum 30° for 2 weeks. After this time, an increased flexion was allowed up to 60° for another 2 weeks, followed by 90° flexion and after 6 weeks up to 120° flexion. Eight weeks after surgery, free range of motion was possible, but still there was a limitation of weightlifting of maximum 1.0 kg, in total for 3 months after surgery. Full weightlifting was allowed half a year after surgery.

#### Follow-up

All three patients left the hospital 1 day after surgery. All were free of pain 6 weeks, respectively 3 months after surgery. In one patient, a new Carpal tunnel syndrome was detected while the 3 months of visitation. This was treated conservatively.

With postoperative physiotherapy, all of them had a good range of motion with full extension seen at the 3-month postoperative consultation. Radiography also showed satisfactory results.

## DISCUSSION AND CONCLUSION

A rupture of the triceps tendon is a very rare entity [2]. Patients often suffered from severe pain at the affected elbow, and there is a loss of muscle strength. The diagnosis is made clinically and general radiography, as well as MRI or ultrasound diagnostic can be used supportive to verify partial ruptures for example. While mostly the ruptures occurred at the point of the triceps tendon insertion at the olecranon, ruptures at the musculotendinous junction are seldom [3, 4]. Total ruptures and partial ruptures with a significant loss of function should be treated with surgery, whereas partial ruptures without significant loss of function can be treated conservatively [2]. Till now, there is no standard surgery technique described in literature. Several reconstruction techniques are described [5].

Among transosseous technique, there is the so called ‘anchor repair’. Horneff *et al.* [6] identified in a multicenter retrospective cohort study the construct of two suture anchors placed medial and lateral in the proximal triceps tendon footprint and one or two distal anchors seated in elbow extension as the most common technique for anchor repair. There is no significant difference in multiple parameters in the final outcome with either technique. The decision to use an anchor or transosseous tunnel repair technique should be based on the surgeon’s preference.

A study by Yeh *et al*. [7] compared biomechanical characteristics of repairs. Conclusion was that anatomic repair with a double-row suture bridge using suture anchors has the least amount of displacement.

Our technique differs from the technique described as common transosseous technique by Horneff and colleagues [6] through two major differences. First, we did not use cruciate tunnels, and second, we did not drill the transverse tunnel. Two parallel tunnels are in our opinion easier to perform and sufficiently cover the footprint and gain a strong connection. This creates a strong connection from the tendon to the olecranon footprint, and intraoperatively there was no gap during flexion of the elbow. Therefore, for us no other tunnel and FiberWire was necessary. With our described cost-effective transosseous technique performed on three patients; all of them were fully satisfied and had an excellent range of motion. No displacement was found during the follow-up time.

## CONFLICT OF INTEREST STATEMENT

None declared.
